# Cultivar-specific responses to organic selenium treatment in tea plants: insights into selenium metabolism and quality traits

**DOI:** 10.3389/fpls.2026.1873674

**Published:** 2026-06-24

**Authors:** Dan Cao, Yanli Liu, Linlong Ma, Xiaomei Yan, Faxing Wan, Xiaofang Jin

**Affiliations:** 1 Fruit and Tea Research Institute, Hubei Academy of Agricultural Sciences, Wuhan, Hubei, China; 2 College of Horticulture and Gardening, Yangtze University, Jingzhou, Hubei, China

**Keywords:** nutrient quality, organic selenium, photosynthetic efficiency, tea plant, tolerance

## Abstract

Foliar application of organic selenium (Se) is a safe and efficient approach to produce Se-enriched tea. Nevertheless, the regulatory mechanisms of organic Se underlying tea plant growth and tea quality remain poorly understood. This study explored the impacts of organic Se on Se accumulation, photosynthetic parameters, metabolite contents, and transcriptome profiles in different tea plant cultivars. Exogenous organic Se markedly elevated leaf Se levels in ‘Zhenong 117’ and ‘Chunyu 1’. Combined transcriptome and metabolome analysis demonstrated that ABC transporters mediated organic Se uptake and accumulation. Key genes (*CGS*, *CTH*) in Se metabolism displayed distinct cultivar-specific expression, driving differential Se metabolic processes. Organic Se improved tea quality by boosting total and umami-related amino acid contents, lowering the polyphenol-to-amino acid ratio, and promoting soluble sugar accumulation via regulating amino acid and sugar metabolic pathways. The two cultivars adopted divergent adaptive strategies, ‘Zhenong 117’ enhanced Se tolerance through activated glutathione metabolism, while ‘Chunyu 1’ adapted by modulating auxin, ethylene, brassinosteroid and jasmonic acid signaling pathways. Overall, cultivar-specific molecular responses determined the differential Se enrichment and quality formation in tea plants under organic Se treatment.

## 1 Introduction

Tea (*Camellia sinensis* (L.) O. Kuntze) ranks among the most economically significant and widely consumed non-alcoholic beverages globally, treasured for its distinctive sensory characteristics and well-documented health properties. The comprehensive metabolic composition of tea leaves, particularly the abundant tea polyphenols and amino acids, determines both the organoleptic quality and the significant health benefits, including antioxidant, anti-senescence, and anti-obesity effects (Cai et al., 2018; Xiong et al., 2017; Sidhu et al., 2024). As a staple cash crop, tea cultivation supports the livelihoods of over 1300 million people worldwide, particularly smallholder farmers, and contributes substantially to the economic development of major producing countries (FAO, 2024). China, as the origin and largest producer of tea, boasts a rich diversity of tea cultivars, but the nutritional quality and economic value of tea leaves are often constrained by environmental factors and agronomic practices. With increasing consumer demand for high-quality and functionally enriched tea products, strategies to enhance tea nutritional value through safe and effective agronomic interventions have garnered widespread attention.

Selenium (Se) is an indispensable trace element for human and animal health, functioning as a catalytic center for essential selenoproteins including glutathione peroxidase and thioredoxin reductase, thereby contributing to antioxidant defense mechanisms, immune regulation, and prevention of various degenerative diseases such as Keshan disease and Kashin-Beck disease (Rayman, 2012; Yang et al., 2020). There is a Se-deficient belt stretching from northeast to southwest China, resulting in 39% to 61% of the population having a daily Se intake below the recommended levels established by the World Health Organization (WHO) and the Food and Agriculture Organization (FAO) of the United Nations (Dinh et al., 2018). Since humans cannot directly assimilate inorganic Se, plants serve as the primary dietary source, converting inorganic Se into bioavailable organic forms such as selenocysteine (SeCys), a selenium-containing amino acid (Trippe and Pilon-Smits, 2021). Tea plants exhibit a natural ability to accumulate and transform inorganic Se into organic forms, with over 80% of Se in tea existing as bioavailable organic Se (Niu et al., 2023). This unique characteristic makes Se-enriched tea a promising functional food for Se supplementation in humans.

The beneficial effects of Se on plant growth and quality formation have been widely confirmed in various crops, including tea plants. Studies have shown that an appropriate concentration of Se can significantly promote plant photosynthesis by increasing chlorophyll content, optimizing the photochemical efficiency of photosystem II (PSII), and regulating the activity of key photosynthetic enzymes such as ribulose-1,5-bisphosphate carboxylase/oxygenase (Rubisco) (Liu et al., 2024; Gao et al., 2024b). Specifically for tea plants, exogenous Se application can effectively increase the accumulation of chlorophyll and carotenoids in leaves, thereby improving the net photosynthetic rate of tea plants and promoting the efficient accumulation of biomass (Li et al., 2021b). In addition to regulating photosynthesis, exogenous Se also has a significant regulatory effect on the accumulation of characteristic flavor compounds in tea, which has become a research focus in recent years. Li et al. (2021b) found that under oxidative stress induced by pesticides, foliar spraying of nano-Se can effectively alleviate the damage of stress to tea quality and improve tea quality by increasing the contents of amino acids such as theanine, glutamic acid (Glu), proline (Pro) and arginine (Arg), and activating the ascorbate-glutathione cycle to reduce the accumulation of malondialdehyde (MDA). Huang et al. (2023) further confirmed that foliar spraying of nano-Se can significantly reduce the contents of total tea polyphenols and catechins, while significantly increasing the contents of total amino acids and theanine, thereby reducing the ratio of tea polyphenols to amino acids and effectively improving the sensory quality of summer tea. In addition, studies by Hou et al. (2025) showed that the synergistic effect of sodium selenite and arbuscular mycorrhizal fungi (AMF) can not only promote tea plant germination, increase plant biomass and photosynthetic pigment content, but also significantly improve the accumulation of Se, free amino acids and L-theanine in tea, while reducing the contents of tea polyphenols, catechins and caffeine (CAF), so as to achieve the comprehensive improvement of tea quality.

Although the regulatory effect of Se on tea plant growth and quality has been extensively studied, there are still obvious deficiencies in current related research. On the one hand, most existing studies focus on the application of inorganic Se sources, while organic Se, with higher bioavailability and lower toxicity, has become a preferred alternative Se source for crop Se biofortification (Yuan et al., 2022), but its regulatory effect on tea plant growth and quality has not been fully studied. Among various Se biofortification approaches, foliar application has demonstrated superior efficiency compared to soil application, as it circumvents soil-related factors including pH, organic matter content, and microbial activity that limit Se bioavailability (Wang et al., 2022). Meanwhile, the specific differences in Se metabolism mechanisms among different tea plant varieties, as well as the internal molecular regulatory network of Se regulating tea plant photosynthetic efficiency and quality formation, are still unclear and urgently need further in-depth exploration.

Therefore, this study aims to explore the effects of foliar application of organic Se (selenomethionine) on Se accumulation, photosynthetic efficiency and quality components of different tea plant varieties. By integrating physiological, biochemical, transcriptomic and metabolomic analyses, this study clarifies the regulatory network underlying the response of different tea plant varieties to organic Se, and provides a theoretical basis for precise Se biofortification strategies, thereby improving tea quality while accounting for genotypic variations in Se metabolism.

## 2 Materials and methods

### 2.1 Plant materials and foliar application

One-year-old cutting seedlings of two tea cultivars, ‘Zhenong 117’ and ‘Chunyu 1’ (*Camellia sinensis* var. sinensis), were used as experimental materials. The seedlings were cultivated in the greenhouse of the Institute of Fruit and Tea, Hubei Academy of Agricultural Sciences (114°33′ E, 30°28′ N, Wuhan, Hubei, China). The tea plants were soil-grown in pots, with a substrate pH of 6.25, containing organic carbon (343.60 g kg^-^¹), total nitrogen (13.65 mg g^-^¹), total phosphorus (2.90 mg g^-^¹), total potassium (16.54 mg g^-^¹), available nitrogen (851.88 mg kg^-^¹), available phosphorus (220.24 mg kg^-^¹), available potassium (3690.00 mg kg^-^¹). The organic Se fertilizer (selenomethionine) was prepared from Se-Met powder. Tea plants with uniform growth and cultivation conditions were divided into three groups according to different Se-Met foliar spray concentrations: control check (CK, sprayed with sterile water), T1 (50 mg/L), and T2 (100 mg/L). Each treatment contained three replicates, with 200 plants per replicate. The experiment was carried out in a greenhouse. The photosynthetic photon flux density was maintained at 300–350 μmol·m^-^²·s^-^¹ for 12 hours daily, with day/night temperatures set at 25 °C/22 °C and relative humidity kept at 80%. All experimental tea seedlings were uniformly pruned on the day of transplanting (20 November 2024). Foliar application of the organic Se-Met solution was carried out at 5:00 p.m. once every 7 days for a total of three applications during April 2025, ensuring complete foliar coverage. On 7 May 2025, the current-year shoots (one bud and two leaves) were harvested and flash-frozen in liquid nitrogen and stored.

### 2.2 Chlorophyll fluorescence parameter determination

Chlorophyll fluorescence parameters were recorded on the first fully-expanded leaves using a Pocket PEA fluorometer (Hansatech Instruments, King’s Lynn, UK) after 30 min of dark adaptation. The parameters included minimal fluorescence intensity at 50 µs with all PSII reaction centres open (Fo), maximal fluorescence intensity with all PSII reaction centres closed (Fm), maximal variable fluorescence calculated as Fm – Fo (Fv), relative variable fluorescence at the I-step (t = 30 ms) calculated as (Fi – Fo)/(Fm – Fo) (Vi), initial slope of the relative variable fluorescence transient reflecting the QA reduction rate (dV/dt_O_), photon energy absorbed per active PSII reaction centre (ABS/RC), excitation energy trapped per active PSII reaction centre leading to QA reduction (TRo/RC), photon flux absorbed per excited cross-section at t = tFm (ABS/CSm), thermal energy dissipation per excited cross-section at t = tFm (DIo/CSm). Other relevant fluorescence parameters are presented in **Supplementary Table 1**. The JIP-test parameters were determined following the method described by Yang et al. (2023).

### 2.3 Determination of quality components in tea leaves

The total polyphenol content was performed according to a previously reported method (Mao et al., 2023). The free amino acids content was determined according to the national standard GB/T 8314-2013. The contents of amino acids, catechins, caffeine and gallic acid (GA) were quantified using high-performance liquid chromatography (HPLC) (Cao et al., 2025; Ma et al., 2025). The soluble sugar content was determined as described previously (Guan et al., 2025). The contents of flavonoids, along with trace elements including Se, phosphorus (P), zinc (Zn), potassium (K), and magnesium (Mg), were quantified in accordance with our previously established method (Cao et al., 2025).

### 2.4 Non-targeted metabolome sequencing and analysis

Non-targeted metabolomics was conducted using a UHPLC-QTOF-MS system (Waters Acquity I-Class PLUS coupled with Xevo G2-XS QTof), with samples collected from CK and T1 treatments. Raw data were acquired with MassLynx V4.2 and processed in Progenesis QI for peak detection, alignment, and annotation against the METLIN database and a custom spectral library. After total peak area normalization, principal component analysis and Spearman correlation were applied to assess within-group repeatability and QC consistency. Metabolites were functionally annotated using Kyoto Encyclopedia of Genes and Genomes (KEGG), Human Metabolome Database (HMDB), and Lipid Metabolites and Pathways Strategy (LipidMaps). Differential metabolites were defined as fold changes (FC) > 1.50, P value < 0.05, and variable importance in projection (VIP) > 1.

### 2.5 Transcriptome analysis and quantitative real-time PCR

Total RNA was extracted from tea plant (under CK and T1 treantments) using the RNAprep Pure Plant Kit and TRIzol Reagent, respectively. RNA quality was verified using a NanoDrop 2000 and an Agilent Bioanalyzer 2100 system. Sequencing libraries were prepared from 1 μg of total RNA using the Hieff NGS Ultima Dual-mode mRNA Library Prep Kit and sequenced on an Illumina NovaSeq platform (150 bp paired-end reads). Raw reads were processed on the BMKCloud platform (www.biocloud.net). Adapters and low-quality bases were removed, and the resulting clean reads were aligned to the *Camellia sinensis* var. *sinensis* (CSS) reference genome (Xia et al., 2020) using Hisat2 (https://daehwankimlab.github.io/hisat2/). Novel transcripts were predicted with StringTie, and gene expression was quantified via FPKM. Differential expression analysis was performed using DESeq2, with thresholds set at adjusted P-value < 0.01 and |FC| ≥ 2. Functional annotation and enrichment analysis of differentially expressed genes were conducted using GO, KEGG and PPI databases. Alternative splicing events were quantified with rMATS, and transcription factors were predicted via TFDB where applicable. Total RNA isolation and subsequent quantitative real-time polymerase chain reaction (qRT-PCR) were performed in accordance with previous relevant studies (Cao et al., 2025). The primers used in this study are shown in **Supplementary Table 2**.

### 2.6 Statistical analysis

All assays were performed in triplicate. Data are expressed as mean ± standard deviation (SD). Statistical comparisons were conducted by one-way analysis of variance (ANOVA), unless otherwise indicated. Differences with P < 0.05 were considered statistically significant.

## 3 Results

### 3.1 Effects of organic Se on chlorophyll fluorescence properties in tea leaves

**Figure 1:**
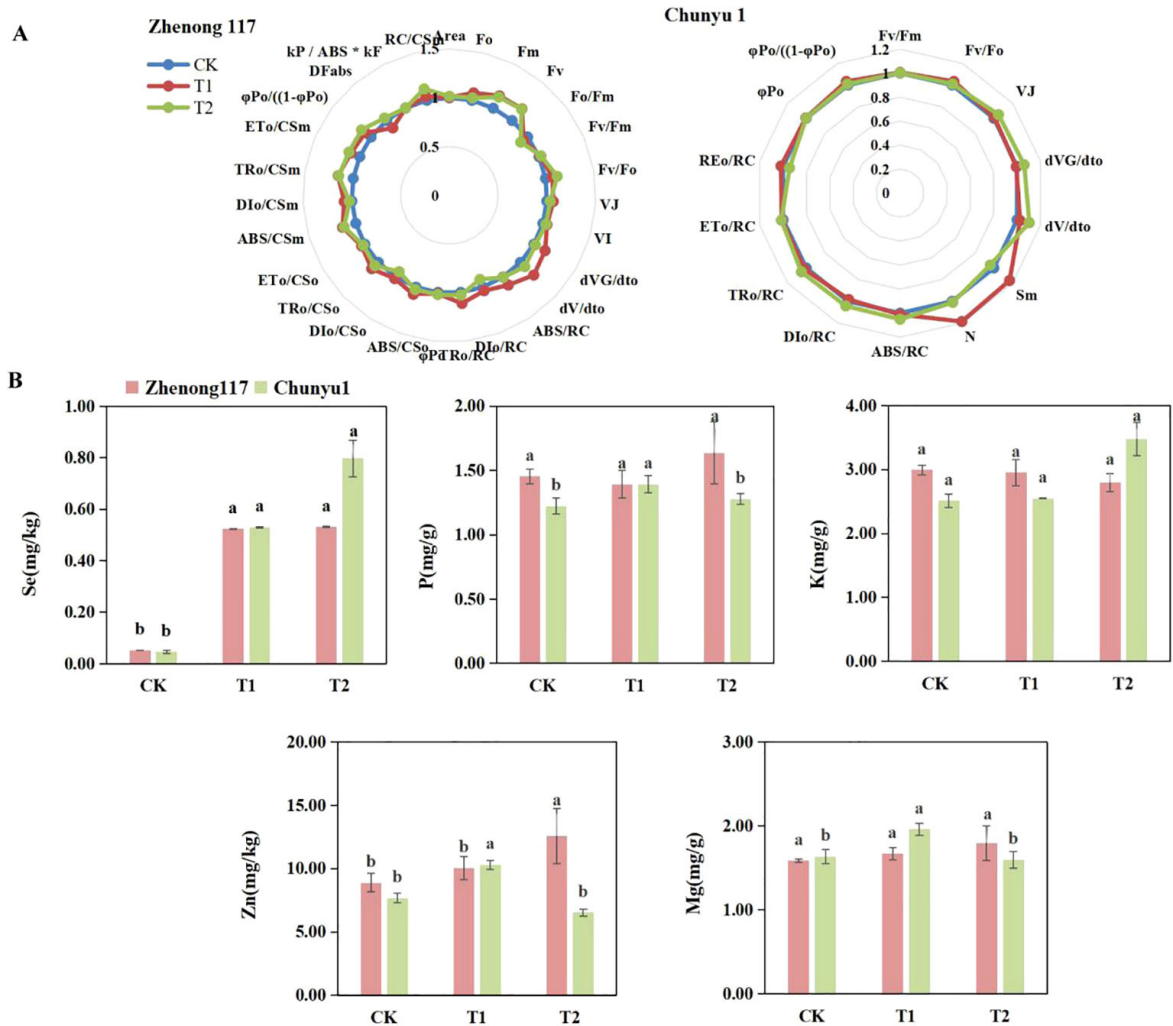
Effects of different concentrations of organic Se treatments on **(A)** chlorophyll fluorescence parameters and **(B)** element contents in tea plant leaves. Different lowercase letters above bars for the same compound indicate statistically significant differences (one-way ANOVA, P < 0.05).

In this study, two tea plant varieties, ‘Zhenong 117’ and ‘Chunyu 1’, were subjected to different Se treatments to assess their impact on photosynthetic parameters. The results showed that photosynthetic parameters (**Figure 1A**), including Vi, dV/dto, ABS/RC, and TRo/RC, significantly increased by 0.92%-8.33%, 6.08%-27.47%, 3.74%-13.09%, and 5.74%-14.35%, respectively, under different treatments, with these differences reaching statistical significance (P<0.05). Particularly for the ‘Zhenong 117’, under treatments T1 and T2, Fm, Fv, ABS/CSm, and TRo/CSm significantly increased by 14.98%-16.54%, 17.31%-17.86%, 8.26%-16.54%, and 17.31%-17.86%, respectively, indicating a notable enhancement in photosynthetic efficiency. Additionally, under treatment T1, the increases in Fo and DIo/CSm were both 11.35%, which were also statistically significant (P<0.05). These findings suggest that exogenous organic Se treatment can significantly improve the photosynthetic efficiency of tea plant leaves, thereby potentially improving tea quality.

### 3.2 Effects of Organic Se on mineral nutrients in tea leaves

As shown in **Figure 1B**, compared with CK, foliar application of organic Se significantly increased the Se content in the leaves. Specifically, in ‘Zhenong 117’, the Se content increased by 8.98 to 9.12 times (P < 0.05), while in ‘Chunyu 1’, the increase in Se content was more pronounced, reaching 10.50 to 16.32 times (P < 0.05). Additionally, to elucidate the impact of organic Se on the absorption of mineral elements by tea plants, this study measured the contents of elements such as Zn, K, P, and Mg in the leaves of different treatment groups. The results indicated that the T1 treatment significantly increased P (13.78%), Zn (34.32%), and Mg (19.89%) contents in the leaves of ‘Chunyu 1’ (P < 0.05). Meanwhile, in ‘Zhenong 117’, the T2 treatment significantly increased the Zn content (41.29%) (P < 0.05). These findings demonstrate that the application of organic Se not only significantly increases the Se content in tea leaves but also has a positive impact on the absorption of other mineral elements.

### 3.3 Effects of organic Se on the main chemical components in tea leaves

**Figure 2:**
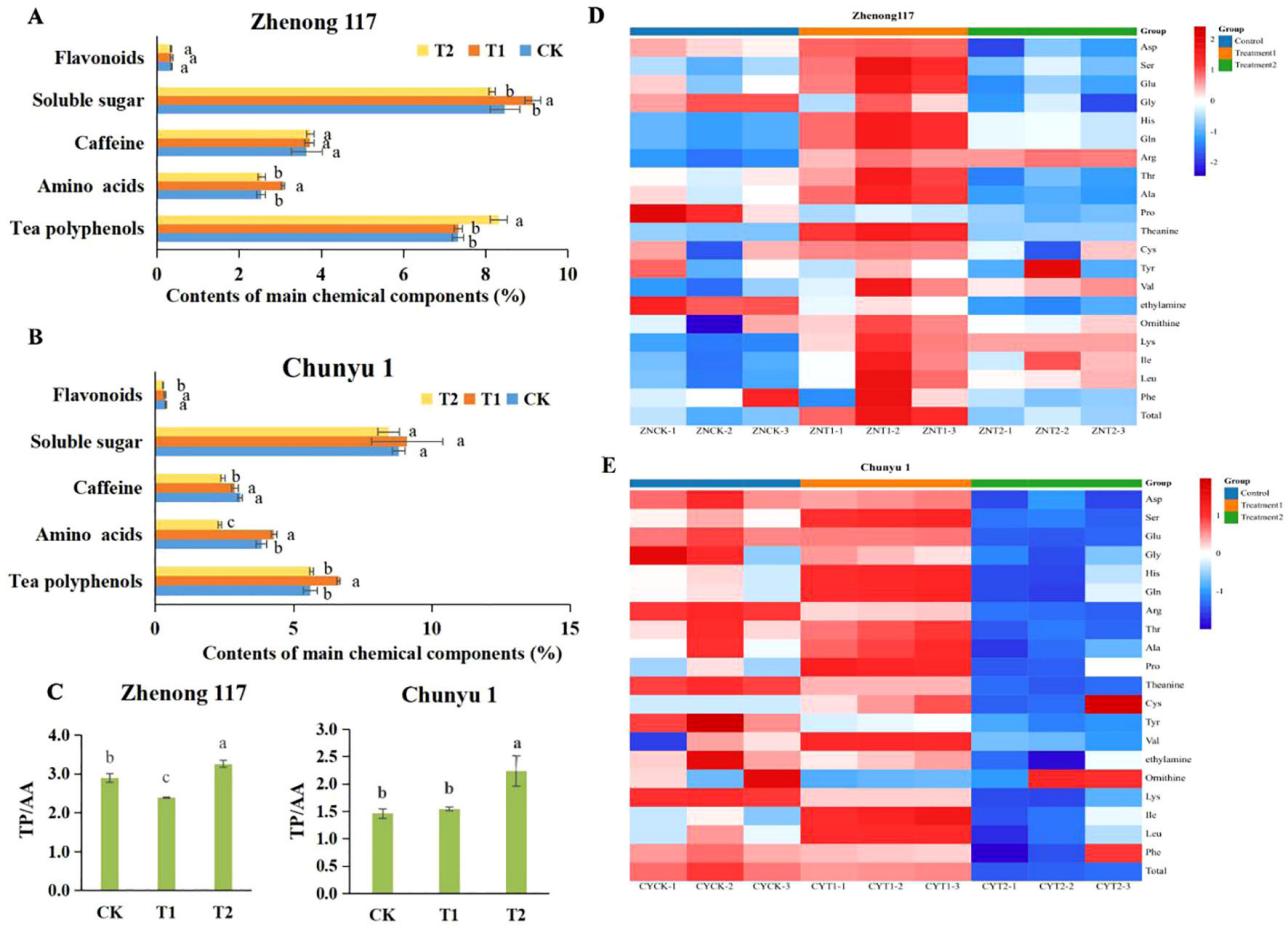
Effects of different concentrations of organic Se treatments on major chemical components in the leaves of ‘Zhenong 117’ and ‘Chunyu 1’. **(A, B)** Changes of tea polyphenols, amino acids, caffeine, soluble sugar, flavonoids in the leaves of ‘Zhenong 117’ and ‘Chunyu 1’. **(C)** Changes of tea polyphenol and amino acid ratio (TP/AA) in the leaves of ‘Zhenong 117’ and ‘Chunyu 1’. **(D, E)** Changes of amino acids in the leaves of ‘Zhenong 117’ and ‘Chunyu 1’ (mean ± SE, n=3, dry weight). Different lowercase letters above bars for the same compound indicate statistically significant differences (one-way ANOVA, P < 0.05). High and low expression levels of genes are colored red and blue, respectively.

As shown in **Figure 2**, under exogenous organic Se treatments at different concentrations, the two tea cultivars—’Zhenong 117’ and ‘Chunyu 1’—exhibited distinct, genotype-specific responses in the accumulation of key secondary metabolites, including tea polyphenols, free amino acids, caffeine, and total flavonoids. Specifically, the tea polyphenol content in ‘Zhenong 117’ was significantly increased by 13.51% (P < 0.05) under the T2 treatment compared to CK.

**Supplementary Figure S1** illustrated the effects of organic Se on catechin content. In ‘Zhenong 117’, T1 treatment significantly increased the content of C by 56.42% (P < 0.05). T2 treatment also led to significant increases in GC (32.87%), EGCG (17.06%), GCG (28.60%), ECG (21.24%), and total catechins (15.60%), all with statistical significance (P < 0.05). Furthermore, GA content was elevated by 29.82% and 20.82% under T1 and T2 treatments, respectively (P < 0.05). In contrast, ‘Chunyu 1’ exhibited the most pronounced enhancement in tea polyphenols under T1 treatment, showing an 18.05% increase (P < 0.05) compared to the control. Regarding free amino acid content, T1 treatment significantly elevated total free amino acids by 21.01% (P < 0.05) in ‘Zhenong 117’, while ‘Chunyu 1’ exhibited a more modest but still significant increase of 11.77% (P < 0.05) under the same treatment. Furthermore, T1 treatment significantly increased the contents of Ser (33.37%), Glu (11.63%), Gln (27.13%), Thr (18.65%), Ala (27.46%), and Theanine (40.79%) in ‘Zhenong 117’ (P < 0.05). T2 treatment also significantly enhanced the Gln content (9.62%) in ‘Zhenong 117’ (P < 0.05). However, under T2, ‘Chunyu 1’ experienced a dramatic 39.14% reduction (P < 0.05) in free amino acids. Further analysis of the ratio of tea polyphenols to free amino acids (TP/AA) revealed that T1 significantly decreased this ratio by 17.41% (P < 0.05) in ‘Zhenong 117’. In contrast, no significant changes in the TP/AA ratio were observed in ‘Chunyu 1’ across all treatments. With respect to soluble sugars, ‘Zhenong 117’ showed a significant 7.94% increase (P < 0.05) only under T1, whereas T2 had no significant effect. ‘Chunyu 1’ exhibited no significant alterations in soluble sugar content under either Se treatment. Caffeine content remained unaffected by both T1 and T2 treatments in ‘Zhenong 117’. Conversely, ‘Chunyu 1’ displayed a significant 20.45% reduction (P < 0.05) in caffeine under T2. Moreover, total flavonoid content was unaltered by T1 in both cultivars. However, T2 induced a significant 27.28% decrease (P < 0.05) in total flavonoids in ‘Chunyu 1’. Coupled with the concurrent declines in free amino acids and caffeine, these results suggest that the T2 concentration of exogenous organic Se may impose mild physiological stress on ‘Chunyu 1’, thereby suppressing the biosynthesis or accumulation of flavonoids and other secondary metabolites. In summary, the modulation of secondary metabolism by exogenous organic Se in tea plants is highly dependent on both application concentration and genetic background. Lower concentrations (50 mg/L) appear beneficial for enhancing quality-related metabolites, whereas higher concentrations (100 mg/L) may trigger adverse physiological responses, particularly in sensitive cultivars such as ‘Chunyu 1’.

### 3.4 Comparative analysis and identification of differential metabolites in two tea cultivars

**Figure 3:**
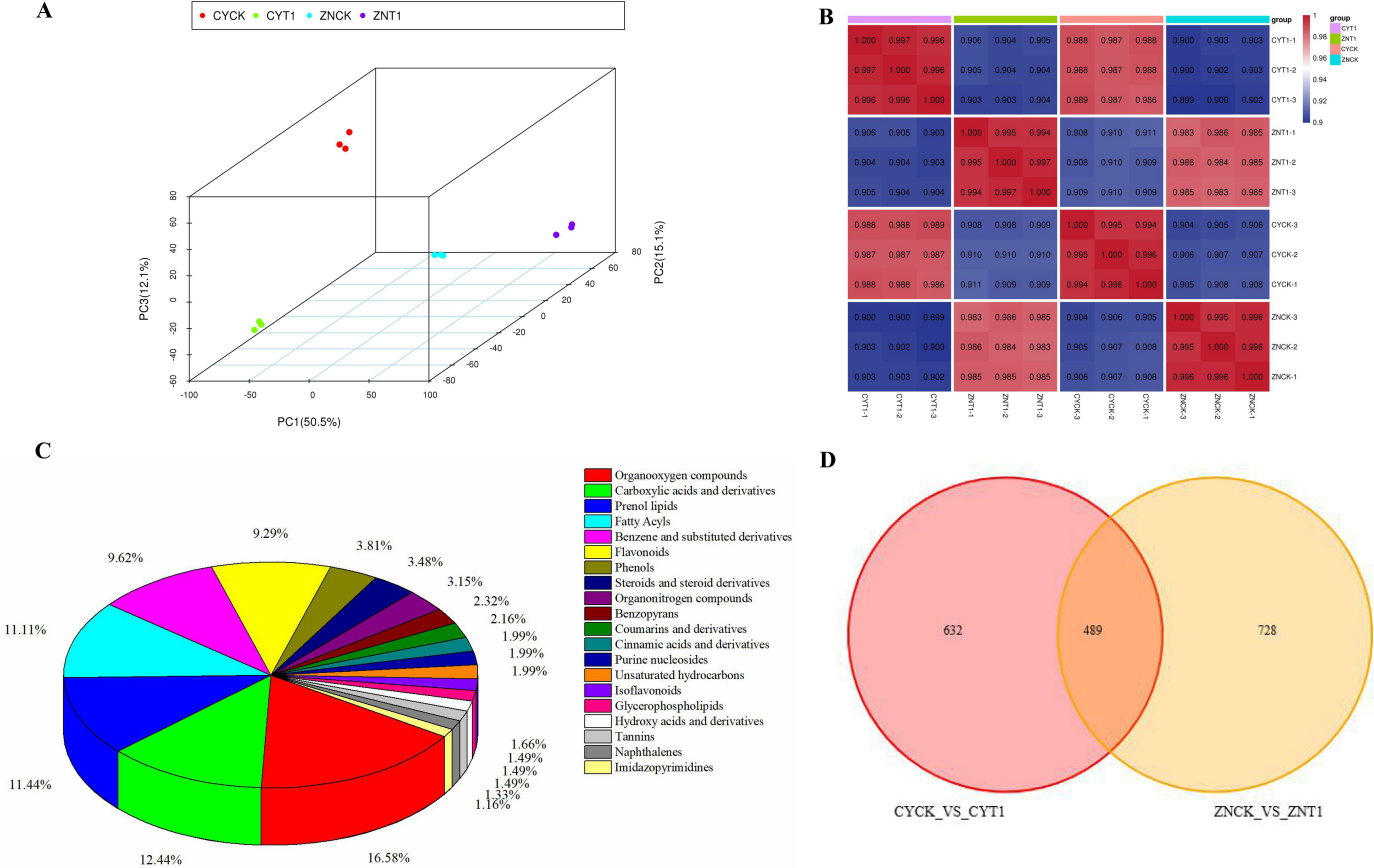
Metabolomic analysis of tea plant leaves. **(A)** PCA analysis. **(B)** Inter-sample correlation heatmap. **(C)** Metabolite distribution. **(D)** Venn diagram of DAMs identified.

Compared with the T2 treatment group, treatment with T1 appeared to facilitate the increase of quality-related metabolites; therefore, the CK and T1 treatment groups were selected for omics analysis. To systematically compare metabolic differences among treatments, an untargeted metabolomic analysis of tea plant leaves was performed using a UHPLC-QTOF-MS platform. A total of 5,095 metabolites were identified across all samples from both cultivars, including 2,493 metabolites detected in positive ionization mode and 2,602 metabolites detected in negative ionization mode. Principal component analysis (PCA) revealed tight clustering of biological replicates within each treatment group, while clear separation was observed between different cultivars and across treatment conditions (**Figure 3A**). This indicates that varying concentrations of organic Se exerted distinct and cultivar-specific effects on the metabolic profiles of the two tea varieties. The correlation heatmap showes high pairwise correlations among the three biological replicates within each treatment group, with tight clustering, demonstrating excellent reproducibility and reliability of the experimental data (**Figure 3B**).

Classification based on chemical structure showed that the six most abundant metabolite classes were: organooxygen compounds (16.58%), carboxylic acids and derivatives (12.44%), prenol lipids (11.44%), fatty acyls (11.11%), benzene and substituted derivatives (9.62%), and flavonoids (9.29%) (**Figure 3C**). Under T1 treatment, a total of 1217 differentially abundant metabolites (DAMs) were identified in ‘Zhenong 117’, of which 567 were upregulated and 650 were downregulated. In ‘Chunyu 1’, 1121 DAMs were detected, including 712 upregulated and 409 downregulated (**Figure 3D**). These results indicate that the two cultivars exhibited distinct metabolic responses to T1 treatment, with ‘Zhenong 117’ exhibiting a more pronounced metabolic perturbation.

KEGG pathway enrichment analysis revealed that distinct tea cultivars exhibited significantly different metabolic profiles under T1 treatment. Specifically, the DAMs of ‘Zhenong 117’ were significantly enriched in metabolic pathways including C5-branched dibasic acid metabolism, taurine and hypotaurine metabolism, and glutathione metabolism under T1 treatment. In contrast, the DAMs of ‘Chunyu 1’ were prominently enriched in arginine and proline metabolism, linoleic acid metabolism, arachidonic acid metabolism, and plant hormone signal transduction pathways (**Supplementary Figure S2**).

### 3.5 Comparative analysis of transcriptional profiles in two tea cultivars

To further investigate the molecular mechanisms underlying the effects of organic Se on tea quality, RNA-seq analysis was performed on 12 samples. A total of 79.21 Gb of clean data were generated, with each sample yielding at least 6.00 Gb. The Q30 base percentage across all samples exceeded 97.49%, indicating high sequencing accuracy and data reliability. Clean reads were aligned to the reference tea plant genome, achieving mapping rates ranging from 87.12% to 89.47%, which reflected robust alignment performance. The inter-sample correlation heatmap and FPKM-based expression distribution boxplot collectively demonstrated high consistency among biological replicates within the same treatment group, confirming the stability and reproducibility of the experimental data (**Supplementary Figures S3A, B**).

Differentially expressed genes (DEGs) were screened using a threshold of FC ≥ 2 and false discovery rate (FDR) < 0.01. The expression profiles of all identified DEGs are shown in the clustered heatmap in **Supplementary Figure S3C**. Compared with the control group of ‘Zhenong 117’ (ZNCK), 1845 (994 up-regulated and 851 down-regulated) DEGs were identified in T1 treatment group of ‘Zhenong 117’ (ZNT1); compared with the control group of ‘Chunyu 1’ (CYCK), 4053 (1532 up-regulated and 2521 down-regulated) DEGs were identified in the T1 treatment group of ‘Chunyu 1’ (CYT1) (**Supplementary Figure S3D**).

**Figure 4:**
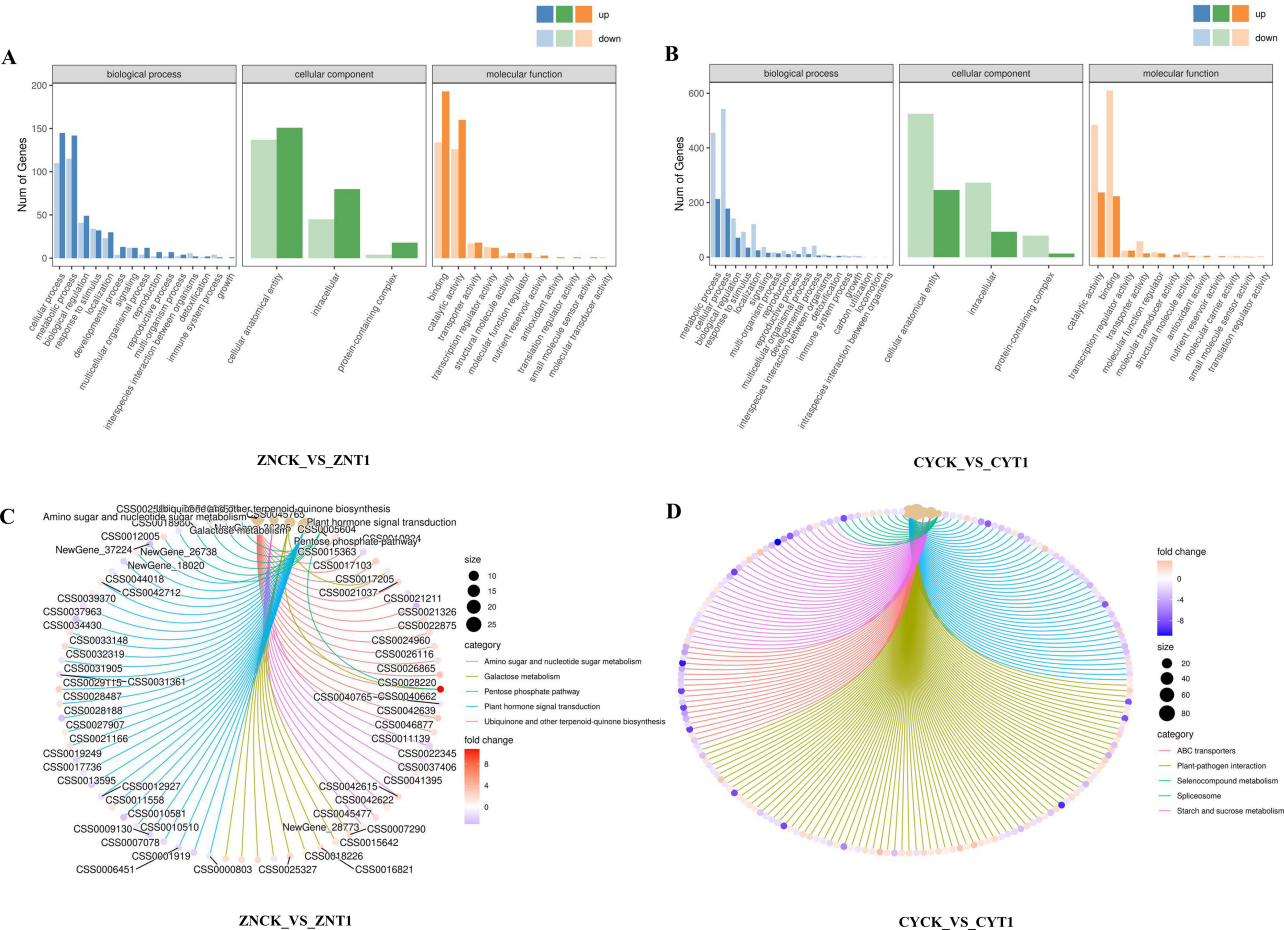
Transcriptomic responses of tea plants to organic Se treatments. **(A)** GO enrichment analysis of DEGs in ZNCK vs ZNT1. **(B)** GO enrichment analysis of DEGs in CYCK vs CYT1. **(C)** KEGG enrichment analysis of DEGs in ZNCK vs ZNT1. **(D)** KEGG enrichment analysis of DEGs in CYCK vs CYT1.

GO enrichment analysis of DEGs was performed across three major categories: Biological Process (BP), Molecular Function (MF), and Cellular Component (CC). Under T1 treatment, within the BP category, ‘Zhenong 117’ showed significant enrichment in pathways such as cellular process and metabolic process, with upregulated genes predominating. In contrast, ‘Chunyu 1’ was also significantly enriched in the same pathways, but with downregulated genes as the majority. In the CC category, both varieties were enriched in terms including cellular anatomical entity, intracellular, and protein-containing complex, with ‘Zhenong 117’ exhibiting predominantly upregulated genes and ‘Chunyu 1’ showing mainly downregulated genes. In the MF category, both varieties were significantly enriched in functions such as binding and catalytic activity, with ‘Zhenong 117’ displaying a pattern dominated by upregulation and ‘Chunyu 1’ showing a pattern dominated by downregulation (**Figures 4A, B**). GO enrichment analysis revealed that ‘Zhenong 117’ exhibited a dominant upregulation of genes across all major enriched pathways, indicating that this cultivar can rapidly activate basal metabolism in response to organic Se treatment and maintain cellular homeostasis through active regulation of cell structure and protein synthesis. In contrast, the enriched pathways in ‘Chunyu 1’ were dominated by downregulated genes, reflecting significant inhibition of early metabolic activities and obvious disturbances to basic cellular structures and protein homeostasis.

By systematically identifying the key metabolic pathways in tea plants in response to organic Se using KEGG enrichment analysis, the results revealed significant differences in the metabolic pathway enrichment characteristics between the two tea plant cultivars, ‘Zhenong 117’ and ‘Chunyu 1’. Under T1 treatment, the DEGs in ‘Zhenong 117’ were predominantly enriched in pathways such as amino sugar and nucleotide sugar metabolism and galactose metabolism, suggesting that these pathways may play a central role in the response of this cultivar to organic Se under T1 conditions (**Figure 4C**). In contrast, the DEGs in ‘Chunyu 1’ were mainly enriched in pathways including ABC transporters and plant-pathogen interaction (**Figure 4D**).

To verify the reliability of the transcriptome sequencing data, 6 DEGs were selected for validation by qRT-PCR. The results (presented as line charts) and the expression profiles obtained from RNA-seq (presented as bar charts) of these 6 DEGs were compared (**Supplementary Figure S4**). The results showed that the expression trends of the 6 DEGs were highly consistent between the two methods, confirming the accuracy and reproducibility of the transcriptome data.

### 3.6 Major pathway analysis

**Figure 5:**
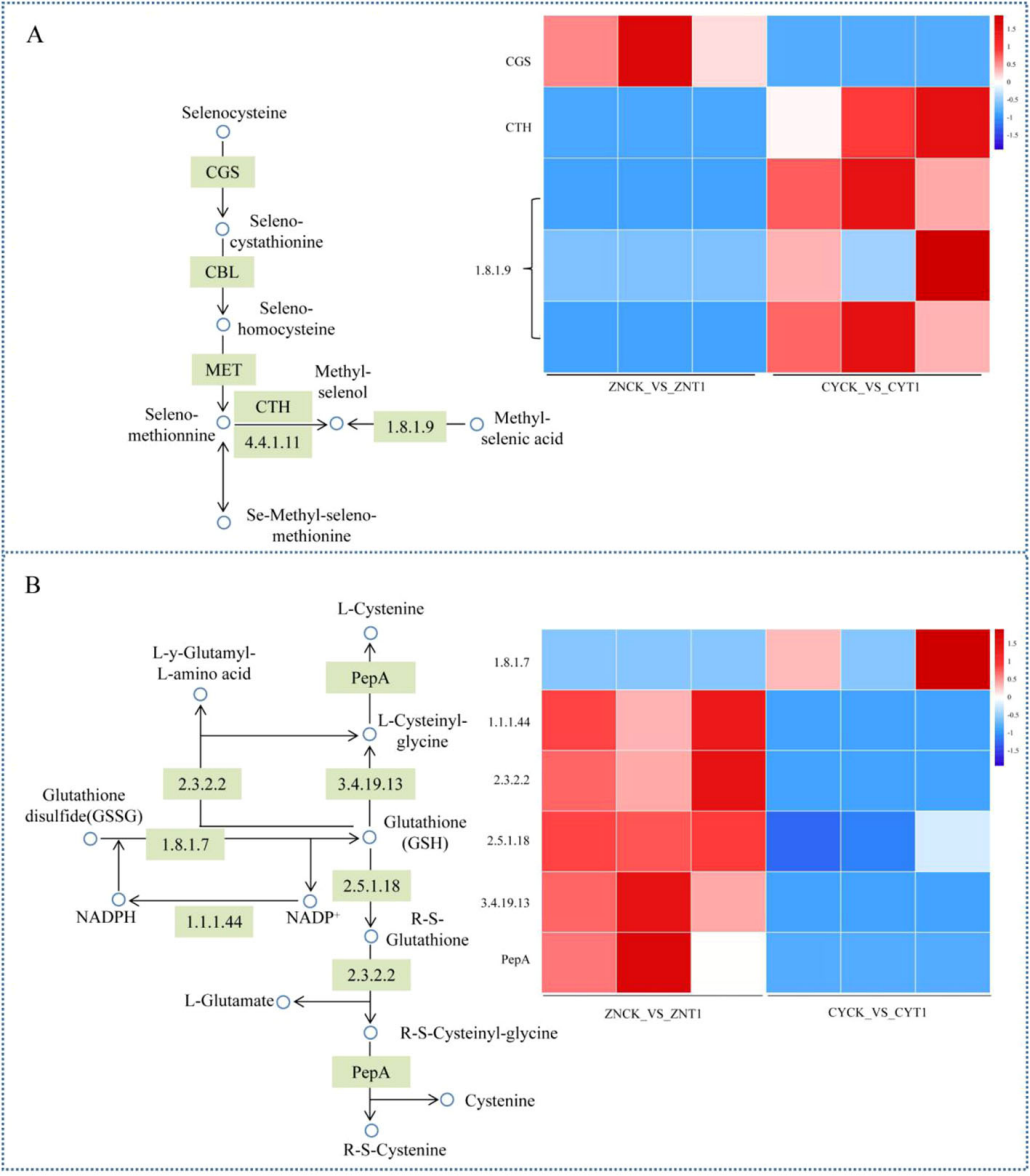
Major metabolic pathways in tea plant leaves under Se treatments. **(A)** DEGs in selenocompound metabolism. **(B)** DEGs are involved in glutathione metabolism.

After foliar application of organic Se, significant differences were observed in the expression of genes related to selenocompound metabolism in the two tea plant cultivars (**Figure 5**). Compared with the control groups (ZNCK, CYCK), the treatment groups (ZNT1, CYT1) showed significant upregulation of several key genes. Specifically, in the ‘Zhenong 117’ treatment group (ZNT1), the expression of the cystathionine γ-synthase gene *CGS* (CSS0019809) was significantly increased. Similarly, in the ‘Chunyu 1’ treatment group (CYT1), the expression of *CTH* (CSS0012078) and 1.8.1.9 (CSS0028147, CSS0040759, CSS0042576) also exhibited a significant upregulation trend (**Figure 5A**).

Following treatment with organic Se (T1), significant changes were observed in the expression of genes related to glutathione metabolism in the two tea plant cultivars (**Figure 5B**). In ‘Zhenong 117’, compared with the ZNCK group, the ZNT1 group showed significant upregulation of glutathione S-transferase gene (*GST*, 2.5.1.18, CSS0036514), 6-phosphogluconate dehydrogenase gene (*G6PD*, 1.1.1.44, CSS0045765), and *PepA* gene (CSS0037781). In ‘Chunyu 1’, compared with the CYCK group, the CYT1 group exhibited significant upregulation of glutathione reductase gene (1.8.1.7, CSS0045927).

### 3.7 Transcriptome–metabolome integration analysis of DEGs and DAMs

**Figure 6:**
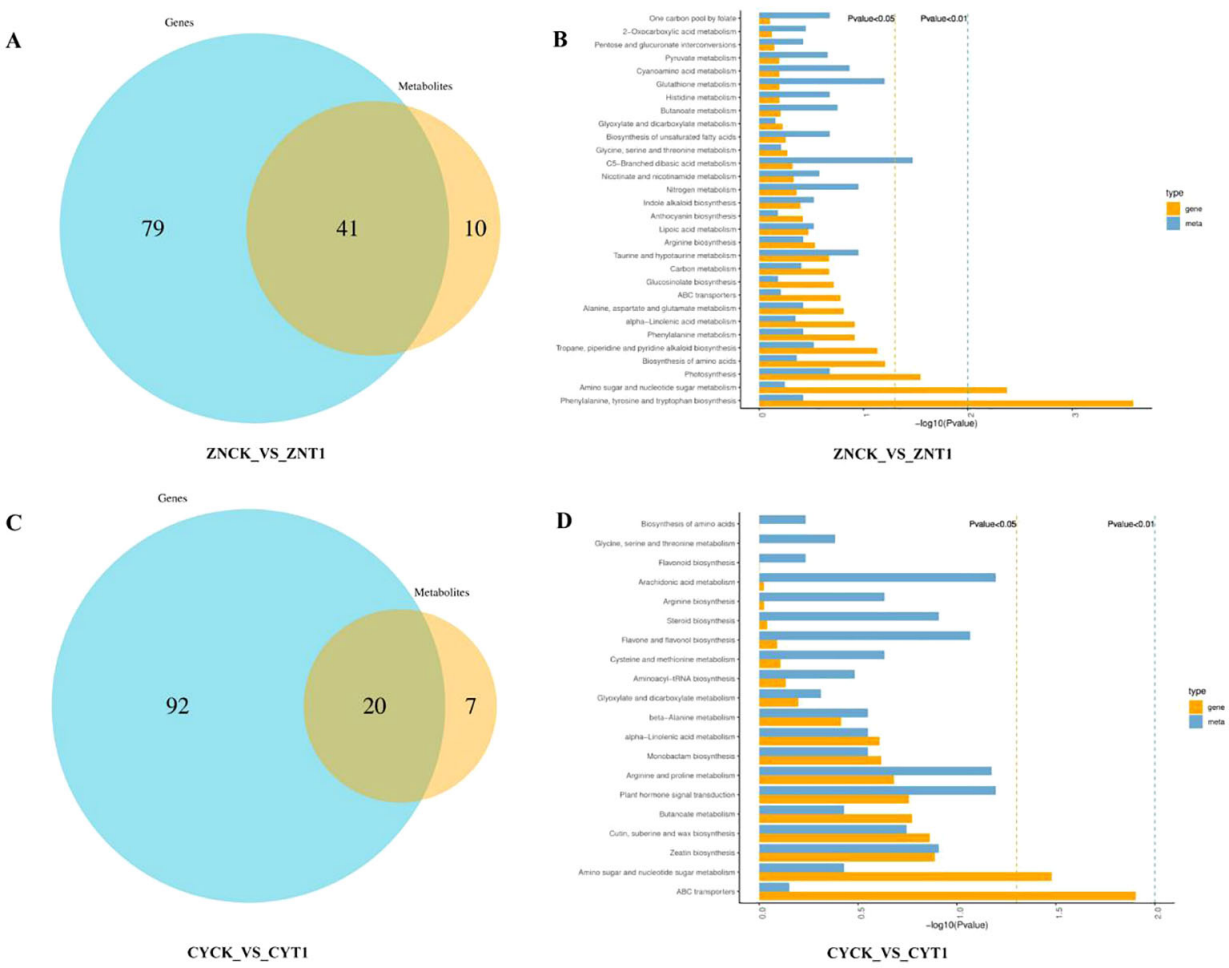
Integrated analysis of DAMs and DEGs in two tea cultivars under T1treatment. **(A, C)** Venn diagram illustrating the number of common and unique pathways. **(B, D)** Common pathway showing the degree of enrichment of DEGs or DAMs.

This study compared the pathway enrichment associated with DEGs and DAMs in two sets of comparisons (ZNCK vs. ZNT1 and CYCK vs. CYT1). In the comparison of ZNCK vs. ZNT1, a total of 41 pathways were co-enriched by DEGs and DAMs (**Figure 6A**), among which the most significantly enriched included “biosynthesis of amino acids”, “photosynthesis”, “nitrogen metabolism”, “glutathione metabolism”, “glycine, serine and threonine metabolism”, “arginine biosynthesis”, “alanine, aspartate and glutamate metabolism” and “ABC transporters” (**Figure 6B**). In CYCK vs. CYT1, 20 pathways were co-enriched by DEGs and DAMs (**Figure 6C**), with the most significant enrichment observed in “biosynthesis of amino acids”, “cysteine and methionine metabolism”, “arginine and proline metabolism”, “flavone and flavonol biosynthesis”, “cutin, suberine and wax biosynthesis” and “ABC transporters” (**Figure 6D**). It is noteworthy that “biosynthesis of amino acids”, “arginine biosynthesis”, “amino sugar and nucleotide sugar metabolism” and “ABC transporters” were consistently significantly enriched in both comparisons.

### 3.8 Integrated analysis of the transcriptome and metabolome of DEGs and DAMs related with biosynthesis of amino acids

**Figure 7:**
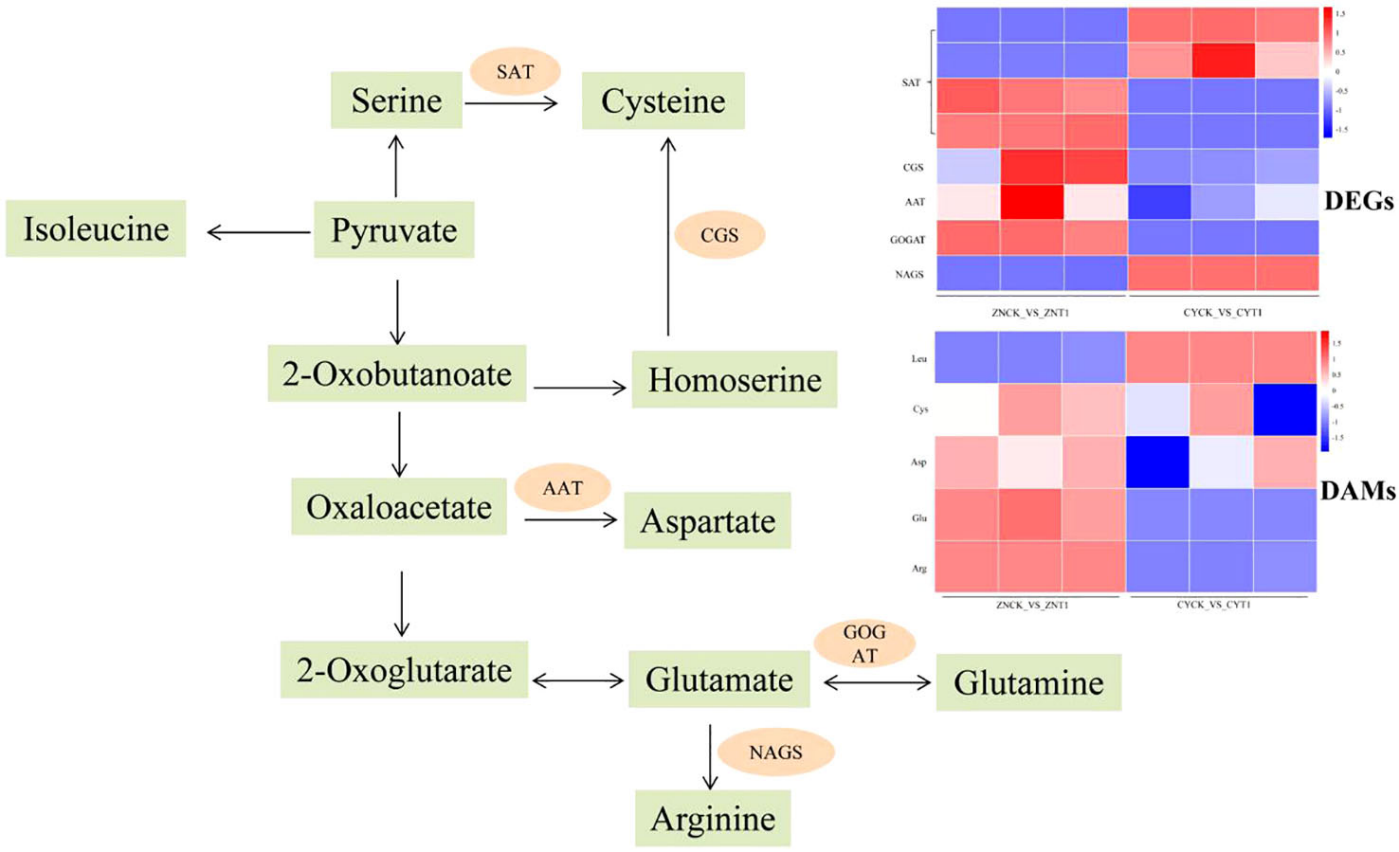
Involvement of DAMs and DEGs in the biosynthesis of amino acids under T1 treatment. Boxes represent metabolites, while circles represent genes. Red arrows indicate upregulated genes/metabolites, and blue arrows indicate downregulated genes/metabolites. The expression levels of genes and metabolites are expressed as Log_2_(FC). High and low expression levels of genes are colored red and blue, respectively.

In the “biosynthesis of amino acids” (**Figure 7**), T1 treatment significantly upregulated the expression levels of key enzyme genes involved in amino acid synthesis, including serine acetyltransferase (*SAT*, CSS0009537, CSS0043861, CSS0026018, CSS0002897), cystathionine gamma-synthase (*CGS*, CSS0019809), aspartate aminotransferase (*AAT*, CSS0019452), and glutamate synthase (*GOGAT*, CSS0050330), while significantly downregulating the expression of the amino-acid acetyltransferase gene (*NAGS*, CSS0044411). Consistent with the trend of gene expression changes, T1 treatment significantly increased the contents of Lys, Asp, and Glu in ‘Zhenong 117’, as well as the contents of Lys and Asp in ‘Chunyu 1’. Meanwhile, it significantly decreased the content of Leu in ‘Zhenong 117’ and the content of Arg in ‘Chunyu 1’.

### 3.9 Integrated analysis of the transcriptome and metabolome of DEGs and DAMs related with ABC transporters

Analysis of DEGs and DAMs enriched in the ABC transporters pathway (**Supplementary Table 3**) revealed distinct characteristics in the gene expression of ABC subfamily members between the two cultivars. In the ABCA subfamily, one ABCA3 member was upregulation in each cultivar, with NewGene_946 in ‘Zhenong 117’ and CSS0007898 in ‘Chunyu 1’. For the ABCB subfamily, ‘Zhenong 117’ showed upregulation of two ABCB1 genes (CSS0007535, CSS0012142) and downregulated of another two ABCB1 genes (CSS0004595, CSS0044182), while only one ABCB1 gene (CSS0012075) was downregulated in ‘Chunyu 1’. In the ABCC subfamily, ‘Zhenong 117’ exhibited a significant upregulation trend, including five ABCC1 genes (CSS0014181, CSS0023565, CSS0024146, CSS0040380, CSS0043095) and two ABCC2 genes (CSS0006975, NewGene_28893), all of which were upregulation; no significant differential expression of genes in this subfamily was detected in ‘Chunyu 1’. Regarding the ABCG subfamily, two members were upregulation in ‘Zhenong 117’, namely SNQ2 (CSS0016744) and ABCG2 (CSS0024996), whereas ‘Chunyu 1’ had one SNQ2 gene (CSS0036685) upregulation and another SNQ2 gene (CSS0046731) downregulated.

### 3.10 Integrated analysis of the transcriptome and metabolome of DEGs and DAMs related with plant hormone signal transduction

In the “plant hormone signal transduction” pathway (**Supplementary Table 4**), T1 treatment significantly regulated the expression of multiple key genes in ‘Chunyu 1’. In auxin signaling, the expression of *ARF* (CSS0007365, CSS0011558) and *GH3* (CSS0001919) genes was significantly inhibited. In cytokinin signaling, the expression of *CRE1* (CSS0011606, CSS0046207), *AHP* (CSS0047346), and *B-ARR* (CSS0008648) genes was also markedly suppressed, accompanied by a marked upregulation of cytokinin content (neg_5279). In gibberellin signaling, *DELLA* (CSS0022566) gene expression was significantly downregulated. In abscisic acid signaling, *PYR/PYL* (CSS0007401, CSS0017736, CSS0027597, CSS0047272) was upregulated. In ethylene signaling, *ETR* (CSS0000918) gene expression was inhibited, while *ERF1/2* (CSS0009309) gene expression was notably upregulated. In brassinosteroid signaling, *BAK1* (CSS0005436, CSS0024180, CSS0024342, CSS0025730, CSS0034152, CSS0035107, CSS0047247) gene expression was upregulated, whereas *TCH4* (CSS0006451) gene expression was downregulated. Additionally, in jasmonic acid signaling, *MYC2* (CSS0003764) gene expression was significantly enhanced.

## 4 Discussion

### 4.1 Organic Se enhanced carbon metabolism in tea plants by increasing light use efficiency

Previous studies have shown that appropriate Se levels can enhance plant light energy capture capacity and conversion efficiency, thereby increasing photosynthesis (Zhang et al., 2014; Sun et al., 2025). In this study, treatment with 50 mg/L organic Se significantly increased the ABS/CSm and TRo/CSm values in ‘Zhenong 117’, indicating enhanced light energy capture. Metabolomic and transcriptomic analyses further revealed that organic Se treatment upregulated the expression of several genes related to light energy capture and electron transport in the leaves, including *PsaE* (CSS0050291), *PsaH* (CSS0022866), *PsaO* (CSS0022105), *PsaN* (CSS0010064), *PetF* (CSS0013949), and *PetH* (CSS0021906). These results provide molecular-level evidence supporting the physiological mechanism by which organic Se enhances light energy utilization efficiency by promoting the expression of photosynthesis-related genes. Photosynthesis serves as the central metabolic pathway for carbon assimilation in plants, and its enhanced activity directly promotes the synthesis and accumulation of carbohydrates. Soluble sugars, as primary metabolites of photosynthesis, serve as indicators reflecting the activity of photosynthetic carbon assimilation. In this study, treatment with 50 mg/L organic Se significantly increased the soluble sugar content in the leaves of ‘Zhenong 117’. Transcriptomic and metabolomic analyses further revealed significant alterations in the carbon metabolism pathway. These results were consistent with findings reported by Huang et al. (2023) and Guo et al. (2025), suggesting that appropriate concentrations of organic Se can enhance photosynthetic efficiency, thereby promoting the accumulation of carbon assimilation products.

### 4.2 Organic Se promoted Se accumulation and metabolism in tea leaves

Foliar application of organic Se has been demonstrated to significantly increase total Se content in plants (Gao et al., 2024b). The present study showed that both 50 mg/L and 100 mg/L organic Se treatments significantly enhanced total Se content in both tea cultivars, with Se concentrations falling within the national standard for Se-enriched tea (0.25–4.0 mg/kg). Furthermore, this study found that organic Se can increase the contents of mineral elements such as Zn, P, and Mg in tea plants.Se and Zn are essential components of various antioxidant enzymes in plants, such as glutathione peroxidase and superoxide dismutase (Dong et al., 2021). Mg is the central atom of the chlorophyll molecule and directly participates in the capture and conversion of light energy during photosynthesis (Chen, 2014). As an essential mineral element, P plays pivotal roles throughout the entire life cycle of plants, ranging from the formation of fundamental biological macromolecules to the regulation of complex physiological metabolism, growth, and development. It is closely associated with growth efficiency, yield formation, and stress tolerance of crops (Wang et al., 2021b). These mineral elements can contribute to the improvement of tea quality through diverse biological pathways.

ABC transporters constitute a critical gene family in plants, which is widely involved in the transmembrane transport of diverse substrates including hormones, metal ions, secondary metabolites, and xenobiotics. They play essential roles in plant growth and development, signal transduction, as well as plant–environment interactions (Rea, 2007; Do et al., 2021).Accumulating evidence has demonstrated that ABC transporters are functionally involved in Se transport and tolerance in plants (Wen et al., 2024; Xiao et al., 2024; Wang et al., 2025; Yang et al., 2026). Specifically, in tea plants, some studies have suggested that ABC transporters are associated with Se sequestration or vacuolar storage (Zheng et al., 2023).Consistent with this regulatory role of ABC transporters in Se metabolism, a study on *Medicago sativa* found that member 36 of the G subfamily was identified as a Se-responsive protein (Wang et al., 2021a). In the present study, the expression of four subfamilies, namely ABCA, ABCB, ABCC and ABCG, was significantly induced by organic Se, which was consistent with the findings of Ren et al. (2022) and Yang et al. (2026). These results suggest that ABC transporters not only participate in the uptake, metabolism and tolerance of inorganic Se (selenate, selenite) and nano-Se in tea plants, but also serve crucial functions in the response and metabolism of organic Se.

In Se secondary accumulator plants, several key metabolic pathways closely related to Se accumulation capacity and Se tolerance (Wang et al., 2018). One of the core pathways involves a characteristic intermediate product—seleno-cystathionine—generated during the conversion of SeCys to selenomethionine (SeMet). The accumulation of this compound can effectively prevent free SeCys from being misincorporated into proteins, thereby avoiding Se-induced protein dysfunction and playing a crucial role in cellular protection (Freeman et al., 2010). Specifically, seleno-cystathionine is synthesized by the condensation of SeCys and O-phosphohomoserine catalyzed by CGS, and then cleaved into homoselenocysteine under the action of cystathionine β-lyase (CBL), which is ultimately methylated to form SeMet, achieving stable storage of Se. In addition, another important branch of Se metabolism takes methylselenol (CH_3_SeH) as a key intermediate. Methylselenol can be further converted into volatile dimethyldiselenide (DMDSe) through specific enzymatic reactions, and released through stomata, forming an important pathway for plant Se detoxification (Gabel-Jensen et al., 2010). This study found that under the same organic Se treatment conditions (T1), the expression of *CGS* gene was significantly up-regulated in ‘Zhenong 117’, indicating that it drove the more efficient synthesis of seleno-cystathionine more efficiently, thereby promoting the accumulation and stable storage of SeMet. In contrast, the cultivar ‘Chunyu 1’ showed increased expression levels of methionine gamma-lyase (*CTH*) and 1.8.1.9, suggesting that its metabolic flux was more inclined to the generation of methylselenol and its subsequent volatile conversion, thereby enhancing Se excretion capacity. Although there was no significant difference in total Se content between the two cultivars, the differences in the expression profiles of their key metabolic genes revealed distinct Se utilization strategies: ‘Zhenong 117’ mainly focused on the efficient fixation and storage of organic Se, while ‘Chunyu 1’ tended to undergo dynamic metabolism and volatile detoxification. The discovery of these metabolic differences between cultivars can provide clear molecular targets for the directional breeding of functional Se-enriched tea tree cultivars.

### 4.3 Organic Se improved tea quality

The quality of tea is mainly determined by its internal biochemical components and their proportions. Tea polyphenols, mainly including catechins, are the primary substances that determine the astringency, bitterness and astringency of tea, and also contribute to strong antioxidant activity (Paiva et al., 2020). Although numerous studies have confirmed that the application of nano-Se or organic Se significantly reduces the contents of tea polyphenols and major catechins in tea leaves (Zhang et al., 2024; Huang et al., 2023), our previous study found that treatment with high concentrations of selenite could increase tea polyphenol content (Cao et al., 2025). The present study found that exogenous organic Se can effectively promote the biosynthesis of both tea polyphenols and catechins, including esterified and non-esterified forms, which is consistent with the previous research finding (Xu et al., 2023). These inconsistent findings may be attributed to factors such as Se form, tea cultivar genotype, and growth environment conditions.

Amino acids, especially theanine, are the core source of the fresh, brisk and sweet tastes of tea, and have a positive effect on relieving stress (Li et al., 2016). This study elucidated that organic Se application promoted the accumulation of umami-related amino acids. Notably, theanine is the most abundant free amino acid in tea and, in coordination with aspartic acid and glutamic acid, contributes to the umami and sweet taste of tea infusion, serving as a key determinant of its overall flavor profile (Ye et al., 2018). The TP/AA ratio is a key indicator for measuring the taste coordination of teas such as green tea; a lower ratio usually means a fresh, brisk and mellow taste, while a higher ratio is often accompanied by a strong bitter and astringent taste (Li et al., 2016). Treatment with 50 mg/L organic Se significantly decreased the TP/AA ratio in ‘Zhenong 117’, which was consistent with the findings of Xu et al. (2023) and Guo et al. (2025). These results suggest that organic Se at this concentration can effectively improve the taste quality of tea leaves.

Transcriptomic and metabolomic sequencing results showed that T1 treatment significantly upregulated the expression of key enzyme genes closely related to amino acid synthesis (*SAT*, *CGS*, *AAT*, *GOGAT*), while downregulating the expression of the *NAGS* gene. *SAT* is a key enzyme in cysteine synthesis; cysteine is a precursor of many sulfur-containing amino acids and plays a vital role in maintaining the balance of amino acid metabolism (Wirtz et al., 2023). CGS is involved in the biosynthesis of methionine, an essential amino acid (Bloch et al., 2021). AAT catalyzes the transamination reaction between aspartate and α-ketoglutarate, promoting the synthesis of Asp and Glu (Li et al., 2023). GOGAT is closely involved in the assimilation of ammonium ions in tea plants and promotes the synthesis of Glu, a typical umami amino acid, and further facilitates the biosynthesis of theanine, a characteristic flavor amino acid unique to tea (Huang et al., 2023). In contrast, NAGS is involved in amino acid catabolism, and its downregulated expression may reduce amino acid decomposition, thereby promoting the accumulation of amino acid products. Consistent with the trend of gene expression changes, T1 treatment significantly increased the contents of theanine, Lys, Asp, and Glu in ‘Zhenong 117’, as well as the contents of Lys and Asp in ‘Chunyu 1’. Asp and Glu are typical umami amino acids, and their increased accumulation directly enhances the umami taste of tea leaves. Lys, as an essential amino acid for humans, not only enriches the nutritional value of tea but also synergistically improves taste coordination. Meanwhile, T1 treatment significantly decreased the content of Leu in ‘Zhenong 117’ and the content of Arg in ‘Chunyu 1’. Leu is a branched-chain amino acid that may contribute to the bitter taste of tea, and its reduction is conducive to alleviating the bitterness of tea infusions. Although Arg is an essential amino acid, it has a slight bitter taste, and its appropriate reduction may further optimize the taste balance of tea, which is consistent with the regulatory role of organic Se in improving tea sensory quality. The changes in gene expression and amino acid content indicated that organic Se (T1 treatment) regulated the biosynthesis and accumulation of amino acids in tea leaves by modulating the expression of key enzyme genes in the amino acid synthesis pathway, thereby promoting the accumulation of umami-related amino acids. This finding is consistent with previous research results (Li et al., 2021b; Huang et al., 2023; Sun et al., 2025), which showed that the application of exogenous Se can regulate the metabolism of nitrogen-containing compounds, affect the synthesis and accumulation of amino acids, and further improve the biochemical component quality and sensory flavor characteristics of tea. The differences in the response of amino acid contents between ‘Zhenong 117’ and ‘Chunyu 1’ may be attributed to the genotypic differences between these two tea cultivars, which lead to variations in their sensitivity and response patterns to organic Se treatment. In addition, treatment with 50 mg/L organic Se increased the soluble sugar content by enriching sugar metabolism-related pathways in ‘Zhenong 117’, which could effectively neutralize the bitterness and astringency of tea infusions, as well as enhance their mellow flavor and sweet aftertaste (Gao et al., 2024a). This finding was consistent with previous research results (Huang et al., 2023; Xu et al., 2023; Sun et al., 2025), further confirming the regulatory role of appropriate concentrations of organic Se in improving tea sensory quality.

### 4.4 Organic Se enhanced tea plant defense responses

In this study, treatment with 50 mg/L organic Se significantly activated the glutathione metabolic pathway in ‘Zhenong 117’, upregulating the expression of *G6PD* and *GST* genes. G6PDH is a key enzyme in the pentose phosphate pathway (PPP), and the NADPH it produces is a critical reducing agent in cells. This reducing power is essential for maintaining the activity of glutathione reductase (GR), which reduces oxidized glutathione (GSSG) to reduced glutathione (GSH), thereby ensuring the continuous regeneration and supply of cellular GSH (Chen et al., 2024). GSH serves as a cofactor for various antioxidant enzymes, such as glutathione peroxidase (GPx). Therefore, the upregulation of *G6PDH* expression might represent an adaptive regulatory mechanism in tea plants to cope with organic Se-induced stress, enhancing PPP metabolic flux to ensure sufficient NADPH and GSH supply. Concurrently, GST, another key component of the glutathione metabolic pathway, can catalyze the conjugation of GSH with potentially toxic Se compounds, forming seleno-glutathione conjugates. This process might reduce their cytotoxicity and facilitate their compartmentalization into organelles like vacuoles, thereby improving Se tolerance in tea plants (Zheng et al., 2023). Previous studies have indicated that GSTs are among the significantly upregulated gene families in response to Se in tea cultivars with strong Se accumulation capacity (Zheng et al., 2023).

As endogenous signaling molecules, phytohormones extensively participate in the regulation of plant growth, development, and stress responses. In this study, treatment with 50 mg/L organic Se significantly downregulated the expression of genes associated with auxin biosynthesis, while concurrently upregulating the expression levels of key genes involved in the biosynthesis pathways of ETH, BR, and JA, thereby activating the “plant hormone signal transduction” pathway. These results were consistent with the findings of Li et al. (2021a). The above findings suggested that exogenous Se might optimize resource allocation strategies in tea plants through a dual mechanism: on one hand, by suppressing auxin-mediated vegetative growth pathways to limit energy consumption; on the other hand, by activating ETH-, BR-, and JA-mediated defense responses to enhance stress tolerance, thereby achieving dynamic balance between growth and defense.

The above results indicated that ‘Zhenong 117’ and ‘Chunyu 1’ exhibited distinct physiological response strategies to organic Se treatment. Specifically, the two cultivars might adapt to Se treatment by activating different metabolic networks or signaling pathways. This cultivar-specific response pattern provides a theoretical basis for explaining the selective improvement effect of organic Se treatment on tea quality components—that is, this treatment tended to improve the quality traits of ‘Zhenong 117’, which might be attributed to its more efficient Se response mechanism or better ability to regulate the growth-metabolism balance.

## 5 Conclusion

In this study, the effects of organic Se on tea quality in different tea cultivars were investigated. The results showed that foliar application of organic Se significantly increased Se content and enhanced photosynthetic efficiency in tea leaves of both cultivars. Treatment with 50 mg/L organic Se effectively improved tea quality by increasing the contents of umami amino acids and sweet soluble sugars, while reducing the content of bitter amino acids. Significant genotypic differences existed in the responses of different cultivars to organic Se, which might be closely related to the activation of distinct metabolic pathways in different cultivars upon organic Se treatment. The results of this study provide a theoretical foundation for the breeding of Se-riched and high-quality tea cultivars.

## Data Availability

The original contributions presented in the study are included in the article/**Supplementary Material**. Further inquiries can be directed to the corresponding authors.
