# Supplementary figures and images for: Cultivar-specific responses to organic selenium treatment in tea plants: insights into selenium metabolism and quality traits
Source: Front Plant Sci. 2026 Jun 24;17:1873674. doi: 10.3389/fpls.2026.1873674 (PMC13344118; doi:10.3389/fpls.2026.1873674)

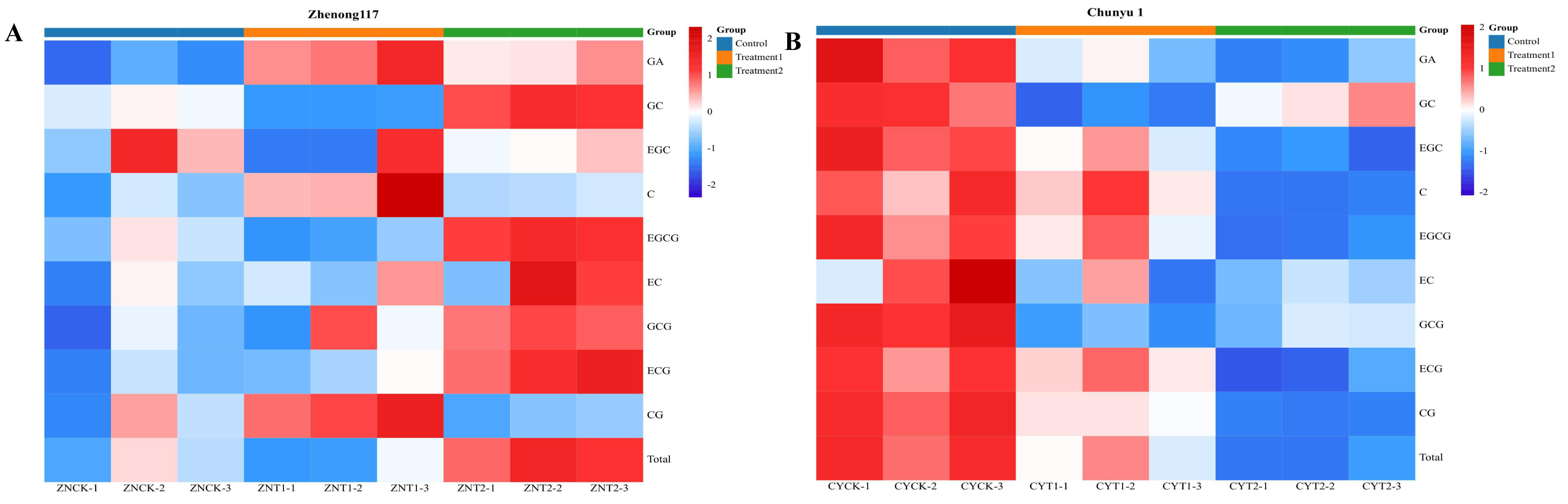

Supplement: Supplementary file 1 [file DataSheet1.zip › Figure S1 Changes of catechin content in the leaves of 'Zhenong 117' and í«Chunyu 1í».tif]

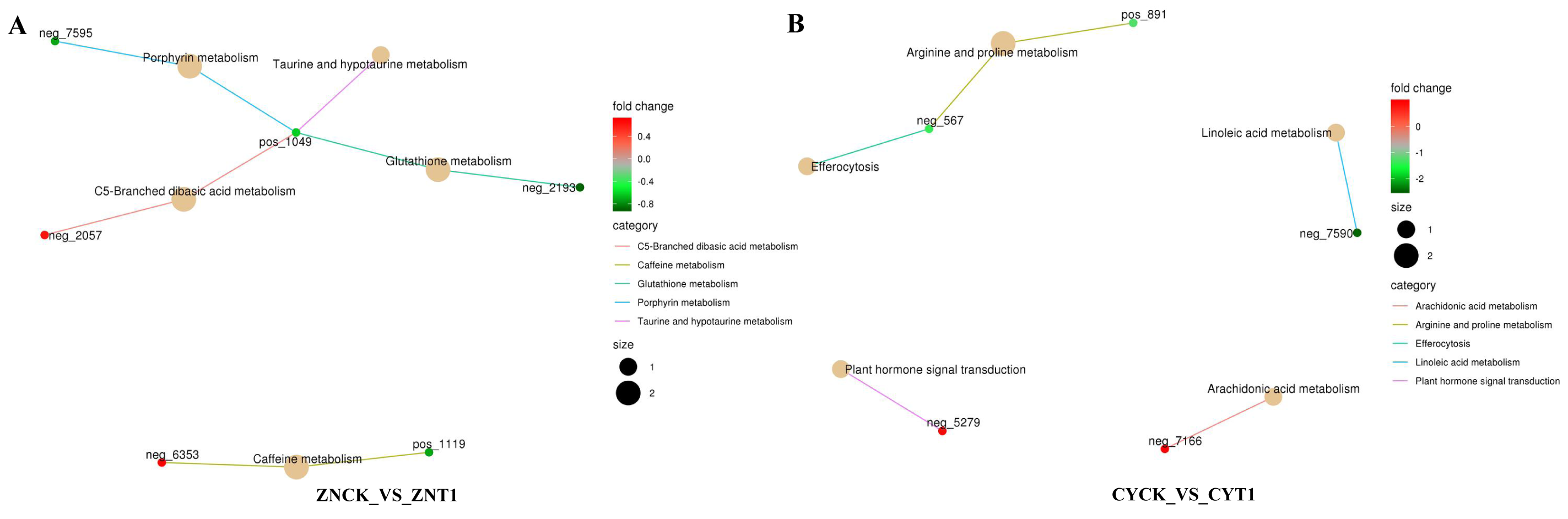

Supplement: Supplementary file 1 [file DataSheet1.zip › Figure S2 Metabolic responses of tea plants to organic Se treatments. (A) KEGG enrichment analysis of DAMs in ZNCK vs ZNT1. (D) KEGG enrichment analysis of DAMs in CYCK vs CYT1..tif]

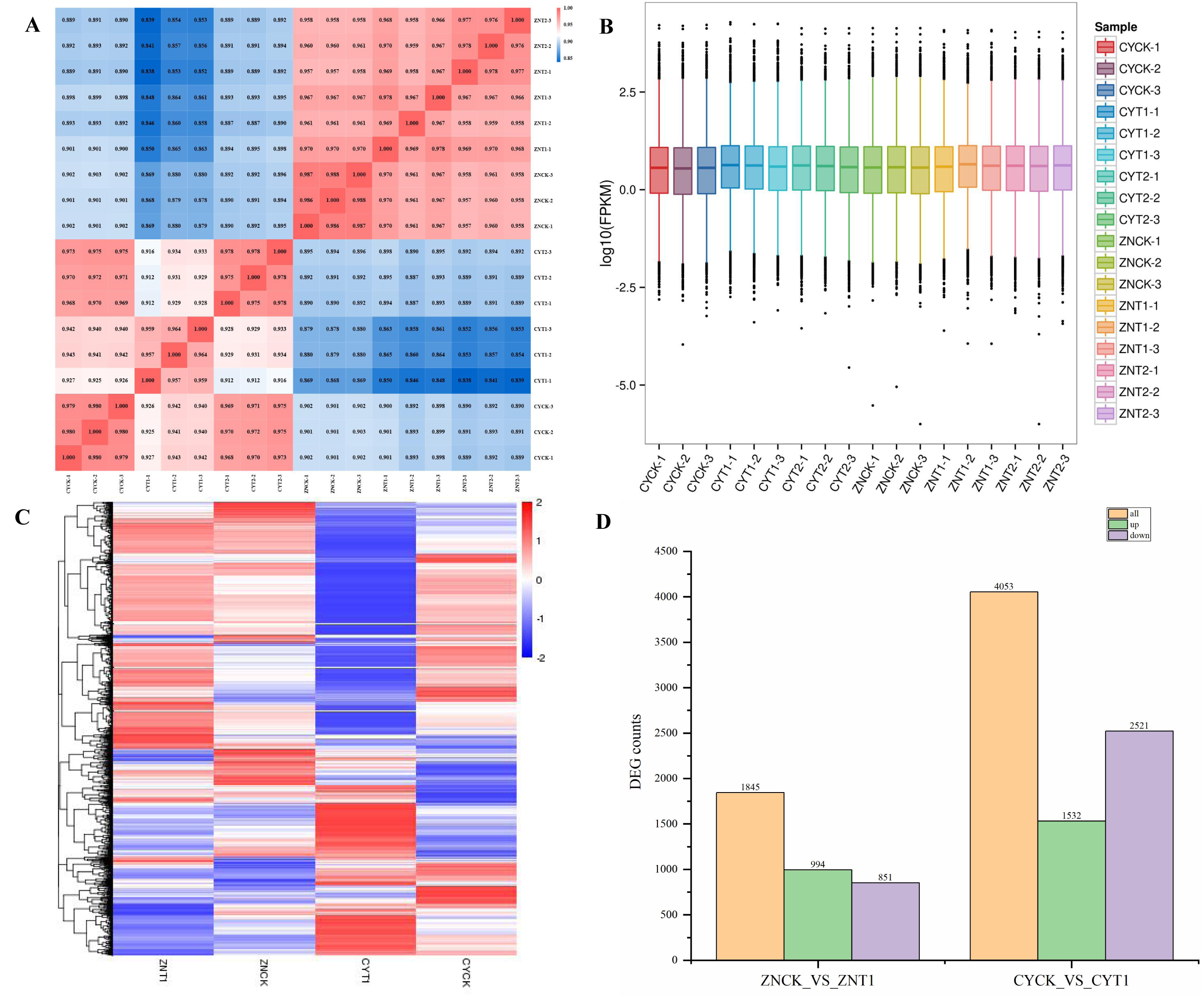

Supplement: Supplementary file 1 [file DataSheet1.zip › Figure S3 Transcriptomic analysis of tea plant leaves.(A) inter-sample correlation heatmap. (B) FPKM-based expression distribution boxplot. (C) Heatmap of DEGs in different treatments. (D) Number of DEGs.tif]

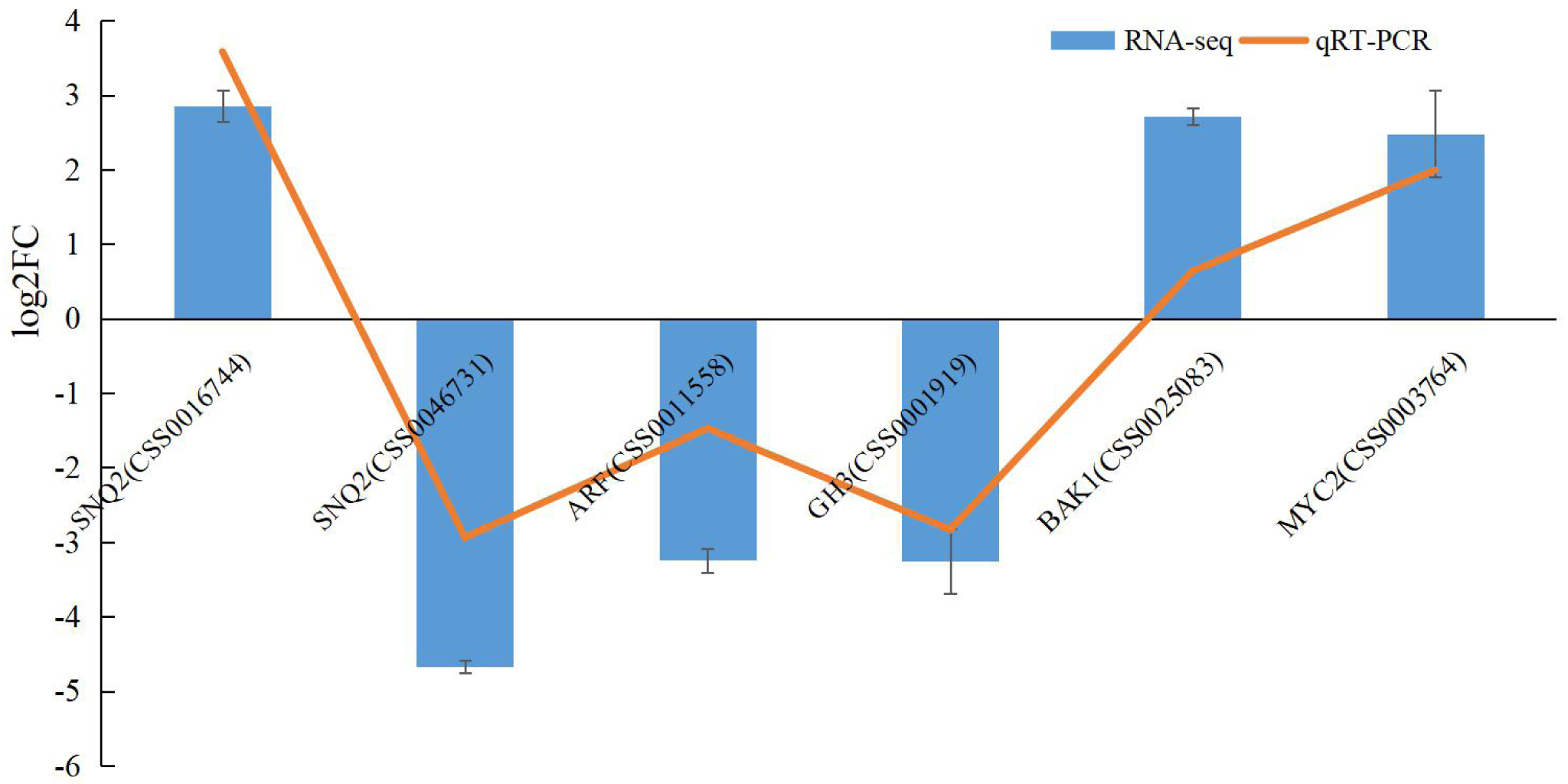

Supplement: Supplementary file 1 [file DataSheet1.zip › Figure S4 qRT-PCR validation.tif]
